# Oral hygiene agents at work: effects on *Streptococcus mutans* and caries risk

**DOI:** 10.3389/fcimb.2026.1768512

**Published:** 2026-02-18

**Authors:** Callahan Katrak, Sydney Reed, Miranda Carter, Malak Khatib, Alexandra Peterson, Kyra Martin, Jessica K. Kajfasz, Jacqueline Abranches

**Affiliations:** Department of Oral Biology, University of Florida College of Dentistry, Gainesville, FL, United States

**Keywords:** chlorhexidine, dental caries, fluoride, hydrogen peroxide, prebiotics, probiotics, *Streptococcus mutans*, zinc

## Abstract

Dental caries remains one of the most prevalent chronic polymicrobial diseases worldwide, driven by acidogenic and aciduric bacteria, most notably *Streptococcus mutans*, that thrive within oral biofilms. Conventional strategies for caries prevention rely on mechanical plaque removal combined with agents that inhibit bacterial growth, disrupt biofilm formation, or enhance enamel remineralization. Here, we synthesize current evidence regarding a range of key agents that are incorporated into modern oral hygiene products. In addition to describing the mechanisms and efficacy of these agents, we describe their distinct biochemical and ecological effects on *S. mutans* metabolism, acid tolerance, and biofilm development. The agents that are relevant in the present day include fluoride, hydrogen peroxide, chlorhexidine, zinc, prebiotics (such as arginine and xylitol), and probiotics. Fluoride remains the cornerstone of caries prevention through its dual effects on enamel fluorapatite formation and inhibition of bacterial glycolysis, while chlorhexidine and hydrogen peroxide provide broad-spectrum antimicrobial activity. Zinc exhibits multifaceted roles in metabolic inhibition and plaque reduction, whereas pre- and probiotics aim to restore ecological balance by favoring health-associated commensal species. Finally, the review highlights evidence supporting combinatorial and synergistic use of these agents, particularly fluoride pairings, which may yield additive or enhanced protective effects. Understanding the molecular mechanisms that drive the efficacy of these compounds and gaining insight into cumulative influence on oral microbial ecology will drive the development of future treatment strategies.

## 1 Introduction

Dental caries is a chronic oral disease of multifactorial etiology that persists as a substantial global health concern, incurring significant economic burdens and detrimentally impacting quality of life. Despite advances in understanding the microbial dynamics that contribute to tooth demineralization and in the availability of oral hygiene products, epidemiological data consistently highlight a high global prevalence of caries, particularly among children (Petersen, 2003; Uribe et al., 2021; Nath et al., 2023; Huang et al., 2024). Dental caries is characterized by demineralization of the enamel and dentin tissues of the tooth, mediated by acid by-products that derive from bacterial metabolism of sugars present in the host’s diet. The delicate balance between demineralization and remineralization, facilitated by salivary buffering and mineral ions, dictates the progression of dental caries.

*Streptococcus mutans* represents the archetype of an acidogenic and aciduric bacterium whose metabolism can disrupt the ecological balance of the oral plaque microenvironment. The pronounced cariogenic potential of *S. mutans* stems from its capacity to colonize the oral cavity, establish robust bacterial biofilms (dental plaque), and produce substantial quantities of organic acids through the fermentation of dietary carbohydrates. The acidification of plaque biofilm that occurs when *S. mutans* has regular access to fermentable sugars results in an environment in which similar caries-associated organisms are favored and in which the commensal organisms associated with health are hindered (Lemos et al., 2019). A critical virulence factor contributing to the pathogenicity of *S. mutans* is the ability to synthesize and secrete extracellular polymeric substances (EPSs), primarily glucans, from sucrose via glucosyltransferases (GtfB, GtfC, and GtfD) (Bowen and Koo, 2011). These EPSs facilitate strong bacterial adherence to tooth surfaces, promote interbacterial adhesion, and stabilize the biofilm matrix, contributing to the acidic microenvironments that are associated with localized enamel decalcification (Koo et al., 2010; Bowen and Koo, 2011; Hwang et al., 2016).

Robust biofilm matrices provide microorganisms with physicochemical protection from host immune responses and environmental stresses, thereby creating a formidable challenge toward maintaining optimal oral health (Bowen et al., 2018). In response to this challenge, a diverse range of active agents have been incorporated into oral hygiene product formulations. Understanding the precise antimicrobial capabilities of these agents against specific oral bacteria, particularly *S. mutans*, is necessary for optimizing the formulation and indication of oral hygiene products. This review aims to critically evaluate the existing evidence regarding the effects of fluoride, hydrogen peroxide, chlorhexidine, zinc, prebiotics (including arginine and xylitol), and probiotics on *S. mutans* growth, biofilm formation, and overall cariogenicity. By synthesizing these findings, our goal is to provide an understanding of their respective contributions to oral health, thereby informing future developments in dental caries prevention and control strategies.

## 2 Anti-caries agents

### 2.1 Fluoride

Fluoride has been the leading preventive treatment for dental caries for almost a century, owing to its inhibitory effects on bacteria such as *Streptococcus mutans* and on its ability to promote enamel remineralization (Manchanda et al., 2022; Zaffarano et al., 2022). Fluoride may occur naturally in (or can be added to) drinking water and is added to hygiene products such as toothpaste, supplements, and mouthwashes. Severe cases of demineralization may warrant in-office applications of higher-concentration fluoride treatments such as varnishes, gels, and foams. Provided in severe cases in which restorative treatment is not feasible (including for children who will lose their primary dentition), silver diamine fluoride is a highly concentrated fluoride treatment that arrests dental caries (Duffin et al., 2022). It is well established that fluoride treatment exerts its anticaries effect by promoting the formation of fluorapatite, which incorporates into hydroxyapatite, the mineral component of tooth enamel. As fluorapatite is more acid-resistant than hydroxyapatite (pKa of 4.5 vs 5.5), enamel enriched with fluorapatite is able to withstand lower pH levels before demineralization occurs, thereby reducing caries incidence (Marquis et al., 2003).

Under caries conducive, low pH conditions, fluoride diffuses into *S. mutans* cells. Intracellularly, fluoride can inhibit key metabolic enzymes such as enolase, a glycolytic enzyme that catalyzes the conversion of 2-phosphoglycerate to phosphoenolpyruvate, a critical step in energy production and acid generation (Curran et al., 1994). Intracellular fluoride also interferes with the aciduricity of *S. mutans* by inhibiting the F_0_F_1_-ATPase proton pump, which exports protons to avoid cytoplasm acidification while acidifying the extracellular milieu (Li et al., 2022). As fluoride is not selective in inhibiting the enolase of *S. mutans* over that of other oral microbes, the ability to inhibit the *S. mutans* proton pump is a key attribute contributing to the anti-caries impact of this mineral. Fluoride has also been shown to inhibit *S. mutans* biofilm accumulation by reducing EPS production (Pandit et al., 2011). The dual-acting nature of fluoride, contributing to the remineralization of enamel and interfering with virulence and metabolism of *S. mutans*, contribute to its status as a particularly powerful anti-caries agent.

### 2.2 Hydrogen peroxide

Hydrogen peroxide (H_2_O_2_) is a reactive oxygen species (ROS) that is widely incorporated into dentifrices and mouthwashes for its antimicrobial and whitening properties. Currently, a variety of oral hygiene products containing H_2_O_2_ are commercially available for oral debridement and whitening, including toothpastes and mouthwashes, with concentrations commonly ranging from 1.5% to 5% (Colgate® Optic white® Pro series stain prevention hydrogen peroxide toothpaste: colgate-palmolive, 2024; ACT whitening Mouthwash, 2025). Although clinical studies with topical H_2_O_2_ mouth rinses (1.5% to 3%) have demonstrated benefits in reducing dental plaque and gingival inflammation as compared to a placebo treatment (Hasturk et al., 2004; Muniz et al., 2020), H_2_O_2_ is not currently indicated for the treatment of dental caries.

Despite the fact that H_2_O_2_ is not marketed as an anticaries agent, laboratory studies have shown that H_2_O_2_ serves as an antagonistic weapon that suppresses caries associated microbes such as *S. mutans*. As a catalase negative gram-positive bacterium, *S. mutans* is highly susceptible to H_2_O_2_. Exposure to H_2_O_2_ results in DNA damage, protein oxidation, and disruption of membrane integrity, which collectively compromise cell viability and competitiveness (Storz and Imlay, 1999; Baldeck and Marquis, 2008; Kreth et al., 2008). Additionally, H_2_O_2_ suppresses *S. mutans* biofilm formation by interfering with quorum sensing, the bacterial cell-to-cell communication mechanism (De Furio et al., 2017). Elevated salivary H_2_O_2_ levels are associated with oral health and reduced caries risk, in part due to their inhibitory effects on *S. mutans* (Zhu and Kreth, 2012). H_2_O_2_ is endogenously produced by immune cells as well as by health-associated oral bacteria such as *Streptococcus sanguinis*, *Streptococcus gordonii*, and *Streptococcus mitis* via the pyruvate oxidase enzyme (Kreth et al., 2005; Zhu and Kreth, 2012; Fujishima et al., 2013; Yang et al., 2013; Redanz et al., 2018). Peroxigenic streptococci are overall more tolerant to the H_2_O_2_ they produce than *S. mutans*, making H_2_O_2_ a good anti-caries candidate (Redanz et al., 2018). Despite the promising findings for the efficacy of H_2_O_2_ against *S. mutans*, to date, there are no published clinical studies evaluating the role of topical H_2_O_2_ in the reduction of dental caries.

### 2.3 Chlorhexidine

Chlorhexidine (CHX) is a broad spectrum bisbiguanide antiseptic. Due to its efficacy at inhibiting bacteria, fungi, and viruses, CHX was initially used as a disinfectant and later introduced into clinical dentistry after the discovery of its potent antiplaque and anti-gingivitis properties (Milstone et al., 2008; Brookes et al., 2020; Poppolo Deus and Ouanounou, 2022). In the United States, CHX is available only by prescription as a 0.12% chlorhexidine gluconate mouth rinse or as a chlorhexidine-thymol varnish (Rethman et al., 2011). The cationic molecule chlorhexidine bisbiguanide can passively diffuse through the bacterial cell wall, then bind to the negatively charged cell membrane. This binding compromises the membrane integrity, thereby increasing membrane permeability, ultimately resulting in the leakage of low-molecular weight molecules and cytoplasmic components. Clinically, CHX treatment is associated with good adherence, as the compound is able to bind to most oral surfaces including teeth, mucus membranes and salivary glycoproteins due to their negative charge, resulting in an activity period of up to 12 hours following application (Poppolo Deus and Ouanounou, 2022). CHX is also used preoperatively to reduce bacterial load and postoperatively following implant or periodontal surgeries to prevent plaque accumulation and promote wound healing when mechanical cleaning is limited (Rethman et al., 2011; Brookes et al., 2020; Poppolo Deus and Ouanounou, 2022). Common side effects of chlorhexidine include altered taste sensation, tongue discoloration, and tooth staining, which may discourage some from using this antiseptic regularly (Brookes et al., 2020).

The efficacy of CHX treatment in reducing *S. mutans*-associated caries remains a topic of debate. Similar to other bacteria, low concentrations (0.02%-0.06%) of CHX are bacteriostatic to *S. mutans*, while higher concentrations (0.12% or more) become bactericidal, when cytoplasmic coagulation and precipitation occur (Karpiński and Szkaradkiewicz, 2015). *In vivo* and *in situ* studies have provided evidence that CHX treatment can specifically reduce the recovery of viable *S. mutans* colonies and diminish biofilm thickness (Bowden, 1996; Ribeiro et al., 2007; Martínez-Hernández et al., 2020). However, a systematic review conducted by the American Dental Association found that use of a chlorhexidine mouth rinse did not result in a significant reduction in coronal caries (Rethman et al., 2011). In the case of root caries, the panel concluded that application of a chlorhexidine-thymol varnish may help reduce incidence in adults and elderly populations (Rethman et al., 2011). Despite its broad antimicrobial properties, chlorhexidine is not FDA-approved for the treatment or prevention of dental caries (Autio-Gold, 2008).

### 2.4 Zinc

The trace metal Zinc (Zn) is an essential nutrient to all domains of life, functioning as a cofactor for critical enzymes (Andreini et al., 2008; Festa and Thiele, 2011; King, 2011; Ganguly et al., 2022). The Zn content in the human body fluctuates throughout the day based on factors including hormones, stress, trauma, infection, diet, and time of day, with estimates of salivary Zn concentrations ranging widely from 0.2 μM to 280 μM (Greger and Sickles, 1979; King, 2011; Lynch, 2011; King and Rousins, 2014; Sejdini et al., 2018). In spite of its essentiality, excess Zn is toxic to bacterial cells due to its ability to bind to non-cognate metalloproteins, thereby impairing their function (mismetallation), and due to interference with uptake of other essential metals, disrupting the critical balance of intracellular metals (Sandström, 2001; Holt et al., 2012; Shafeeq et al., 2013; Djoko et al., 2015; Lonergan and Skaar, 2019). Due to its antimicrobial and immunomodulatory characteristics along with its low toxicity to mammalian tissues, Zn has been incorporated into oral health products for decades, including over-the-counter toothpastes and mouthwashes. The concentration of Zn in such products ranges from 30 to 150 mM, and administration has shown to lead to several hours of elevated levels of Zn in the oral cavity (Harrap et al., 1984; Lynch, 2011; Fatima et al., 2016; Bowen et al., 2018; Delgado et al., 2018; Rahman et al., 2019). Supplemental Zn treatment has been marketed to improve numerous oral conditions, including formation of healthy enamel, reduction of halitosis, prevention of dental calculus formation, and bolstering of periodontal health (Khajuria et al., 2018; Uwitonze et al., 2020).

Despite the documented benefits of Zn as an antimicrobial agent, the role of this metal in the prevention of dental caries remains a subject of debate. Multiple *in-vivo* studies have found Zn treatment to be ineffective in altering dental plaque composition, plaque pH, or caries outcomes (Compton and Beagrie, 1975; Stephen et al., 1988; Giertsen, 2004; Parkinson et al., 2018). At the bacterial level, advances have been made in understanding the effects of Zn toxicity. For example, Zn treatment has been shown to reduce metabolic activity of cariogenic bacteria *in vitro*, inhibiting acid production by mutans streptococci and inhibiting ATPases, PTS (phosphoenolpyruvate:sugar phosphotransferase system) activity and alkali production (He et al., 2002; Phan et al., 2004). Recent work demonstrated that among oral streptococci, *S. mutans* demonstrates a high tolerance to Zn due to its unique zinc exporter, ZccE. *In vivo* studies using a rodent model showed that daily treatment with Zn modestly inhibited *S. mutans* oral colonization, but a mutant strain lacking ZccE was highly susceptible to Zn treatment, resulting in a significant impairment in oral colonization (Ganguly et al., 2022). Thus, combinatorial approaches with Zn and compounds that target ZccE activity hold promise in eliminating the advantage that *S. mutans* holds over other oral microbes when exposed to excess Zn.

### 2.5 Prebiotics

Prebiotics were first defined by Gibson & Roberfroid in 1995 as “nondigestible food ingredients that beneficially affect the host by selectively stimulating the growth and/or activity of one or a limited number of bacterial species already resident in the colon, and thus attempt to improve host health” (Gibson and Roberfroid, 1995). In 2017, the International Scientific Association for Probiotics and Prebiotics (ISAPP) redefined prebiotics as “a substrate that is selectively utilized by microorganisms conferring a health benefit” encompassing non-food substances and effects beyond the gastrointestinal tract (Gibson et al., 2017). Recent years have seen the incorporation of prebiotics into dental care products to improve oral health and to potentially supplement or replace fluoride (Suresh et al., 2021; Luo et al., 2024). Candidate prebiotics include oligosaccharides, inulin, amino sugars, sugar alcohols, arginine, urea, and nitrates, as all of these molecules exhibit the potential to inhibit caries-associated microbes or to promote the growth of beneficial oral commensal species (Koopman et al., 2015; Zeng et al., 2016; Chen et al., 2020; Agarwal et al., 2022; Zeng et al., 2022). A growing number of conventional and natural-product-based oral hygiene products now contain prebiotics, though their efficacy remains under investigation (Suresh et al., 2021). Among these, sugar alcohols and arginine are the most included compounds (Zheng et al., 2017; Alhumaid and Bamashmous, 2022).

Sugar alcohols, such as erythritol, maltitol, sorbitol, and xylitol, have been researched for their inclusion in oral health products due to their properties as prebiotics, caries inhibitors, and non-cariogenic sugar substitutes (Runnel et al., 2013; Thabuis et al., 2013; Falony et al., 2016; Cocco et al., 2017; Rafeek et al., 2019). Xylitol, in particular, has been extensively studied and included in a wide range of dental products, including toothpastes, mouthwashes, chewing gums, and candies (Alhumaid and Bamashmous, 2022). In *S. mutans*, xylitol has been shown to interfere with PTS activity, thereby inhibiting acid production and becoming toxic once it accumulates in the cytoplasm of oral bacteria. Importantly, clinical studies showed that xylitol can reduce the abundance of some cariogenic oral streptococci in saliva while not affecting the prevalence of commensal streptococci who may be unable to metabolize xylitol (Trahan et al., 1985; Makinen et al., 1995; Trahan et al., 1996; Bahador et al., 2012; Runnel et al., 2013) (Soderling and Pienihakkinen, 2020). Despite the poisoning effect that xylitol exerts on *S. mutans* by interfering with PTS activity, recent work has shown that some gut microbes are able to metabolize xylitol; the impacts of this sugar alcohol on the short chain fatty acid composition of gut microbes is likely to have implications in overall health of the host, further expanding the impact of this prebiotic (Xiang et al., 2021).

Arginine is another widely studied prebiotic compound that has been added to oral care products (Zheng et al., 2017; Wolff and Schenkel, 2018; Luo et al., 2024). Arginine favors the metabolism and competitiveness of commensal bacteria in the oral microbiome by producing ammonia, which neutralizes the acids produced by *S. mutans* in the oral cavity (Agnello et al., 2017; Huang et al., 2017). Also, arginine itself inhibits growth, metabolism and proteins important for *S. mutans* virulence (Chakraborty and Burne, 2017). Clinical studies have shown that arginine supplementation negatively affects dental plaque build-up and disrupts established cariogenic bacterial biofilms (Sharma et al., 2014; Huang et al., 2017; Gloag et al., 2021) while also inhibiting growth of *Candida*, a fungus that interacts synergistically with *S. mutans* in cariogenic conditions (Koopman et al., 2015).

### 2.6 Probiotics

Collectively the human oral cavity can host over 700 potential bacterial species, with any given individual typically harboring between 100–200 species (Dewhirst et al., 2010). When oral microbial homeostasis is disrupted, cariogenic organisms such as *S. mutans* can become overabundant, leading to increased biofilm accumulation, reduced oral pH, and elevated caries incidence (Lemos et al., 2019; Spatafora et al., 2024). Probiotics are considered to be live microorganisms that, when administered directly to the intended environment, provide a health benefit to the host (Gibson et al., 2017). While frequently used for treatment of gut microbiome dysbiosis, probiotic use in the oral environment is a relatively new practice that continues to evolve and improve. In the oral environment, beneficial commensal streptococci are known for their ability to produce hydrogen peroxide and are therefore often included in oral probiotic formulations (Redanz et al., 2018) (Burton et al., 2013b). Several *Lactobacillus* species have demonstrated the ability to impair *S. mutans* biofilm formation via suppression of the genes coding for Gtfs (*gtf*) (Wasfi et al., 2018). Clinically, the effects of *L. reuteri* chewable tablets were shown in one study to reduce caries incidence in children with mixed dentition (Stensson et al., 2014). Unlike the other agents discussed in this review, probiotics are often marketed separately from existing oral hygiene products, such as dentifrices and mouth rinses, and at the current time, relatively few of these products are available on the market or have undergone extensive clinical testing. As such, probiotic supplements may not be monitored for safety, as the Generally Regarded as Safe (GRAS) label, designated by the Food and Drug Administration (FDA), applies only to substances added to food (Burdock, 2000).

Clinical studies examining probiotic efficacy in children with early childhood caries (caries in children in primary dentition) have thus far yielded conflicting results (Di Pierro et al., 2015; Hasslöf et al., 2022; Staszczyk et al., 2022). However, some promising oral probiotic candidate organisms emerged from these studies. For example, treatment with *Streptococcus salivarius* via a lozenge decreased *S. mutans* prevalence after 3 months in caries-active children (Burton et al., 2013a). Alternatively, a blend of strains of *Streptococcus uberis*, *Streptococcus oralis*, and *Streptococcus rattus* provided as chewable tablets decreased overall caries incidence with consistent use after 3 months (Hedayati-Hajikand et al., 2015). Notably, these effects appeared to be dependent on the ability of the probiotic organism to colonize the treated environment, which may prove challenging in cases where *S. mutans* is already established. If treatment is pursued early in life, a child’s oral microbiome may be more receptive to the introduction of beneficial organisms before the community eventually stabilizes as the subject ages. Adult oral microbiomes are typically more stable, and beneficial effects of probiotic treatment may require additional supplementation to favor their implantation in the microbiome, as short-term treatment may result only in temporary colonization (Petersson et al., 2011; Romani Vestman et al., 2015). Ultimately, while oral probiotics show potential for caries prevention, their long-term efficacy remains uncertain as the microbes may have requirements (e.g. nutritional) that must be provided to exert their beneficial function. Further research will provide guidance regarding how to sustain probiotic benefits and to identify strains with consistent colonization and cariostatic properties (Lopes et al., 2024; Garcia et al., 2025).

## 3 Discussion: Combinatorial therapies

A common theme among many of the agents discussed above is that *in-vitro* activity against *S. mutans* was often promising, shedding light on the mechanisms of action that lead to inhibition of the microbe. However, these same treatments often fell short in the clinical setting. Investigations of combination therapy are the next logical step. Not only does evidence point toward combinatorial therapy conferring advantages beyond the individual agents, but real-world oral hygiene behaviors dictate that individuals routinely utilize a combination of these therapeutics in their oral hygiene routine. Administration of these agents can occur either in individual products which contain multiple active agents or through combinatorial use of multiple products, such as dentifrices, mouthwashes, chewing gums, and varnishes.

Due to its prevalence in oral hygiene products, combinatory therapy utilizing fluoride has been the most extensively explored to date. Investigation of the combination of hydrogen peroxide and fluoride has been limited to a single clinical study showing reduced decalcification following use of a mouthrinse incorporating both H_2_O_2_ and fluoride, as compared to the fluoride-only mouthrinse (Boyd, 1992). *In vitro* studies have shown that pairing chlorhexidine with fluoride significantly reduces abundance of *Streptococcus mutans* in multi species biofilms compared to either individual treatment (Erdem et al., 2012). Further, varnishes mixing chlorhexidine and fluoride have shown some efficacy in arresting or slowing root carious lesions in older adults over a 12-month period (Slot et al., 2011; Park et al., 2022). In the case of zinc combination treatment, *in vitro* work has shown the efficacy of zinc and fluoride to reduce the acidogenicity and biofilm formation of *S. mutans* (Izaguirre-Fernandez et al., 1989; Koo et al., 2006). However, a multi-year clinical study of dentifrices with zinc citrate and various fluoride concentrations demonstrated no improvement over their fluoride-only containing counterparts, again highlighting the need to experiment with different combinatorial therapies (Stephen et al., 1988; Josic et al., 2024).

Oral hygiene products that contain both fluoride and a prebiotic agent, either arginine or xylitol, have been the subject of extensive *in vitro* and *in vivo* research. At least six clinical studies have investigated the combined effect of fluoride and arginine, and have shown that together, these agents can reduce caries formation more effectively than fluoride-only control groups (Kraivaphan et al., 2013; Souza et al., 2013; Srisilapanan et al., 2013; Yin et al., 2013; Li et al., 2015; Zheng et al., 2015; Wolff and Schenkel, 2018; Bijle et al., 2019; Yin et al., 2025). *In vitro* work has also shown that when administered to a multi-species biofilm composed of *S. mutans* and a commensal species, the combination of fluoride and arginine was more effective in reducing *S. mutans* biofilm formation than when the agents were tested alone (Zheng et al., 2015). Similarly, although less thoroughly investigated, fluoride in conjunction with xylitol has also shown promise as a combination therapy. Treatments with both fluoride and xylitol were shown to impede the ability of *S. mutans* to produce acid *in vitro*. Clinical studies showed that fluoride and xylitol reduce the abundance of *S. mutans* in the oral cavity following short-term use, and, after 3 years of treatment, to improve decayed, missing, and filled tooth scores (Sintes et al., 1995; Maehara et al., 2005; Arunakul et al., 2011; Bahador et al., 2012).

A great interest in developing ecological approaches to prevent and control caries has emerged in the past decade. For example, lozenges containing arginine and two probiotic species effectively reduced caries incidence and progression in children (Porksen et al., 2023). Together this encouraging result suggests that combining prebiotics with probiotics offers more effective disease prevention and control than either strategy applied alone, likely because the prebiotic can support the probiotics by enhancing their specific activities in disarming or displacing harmful cariogenic microbes and restoring microbial homeostasis.

**Figure 1:**
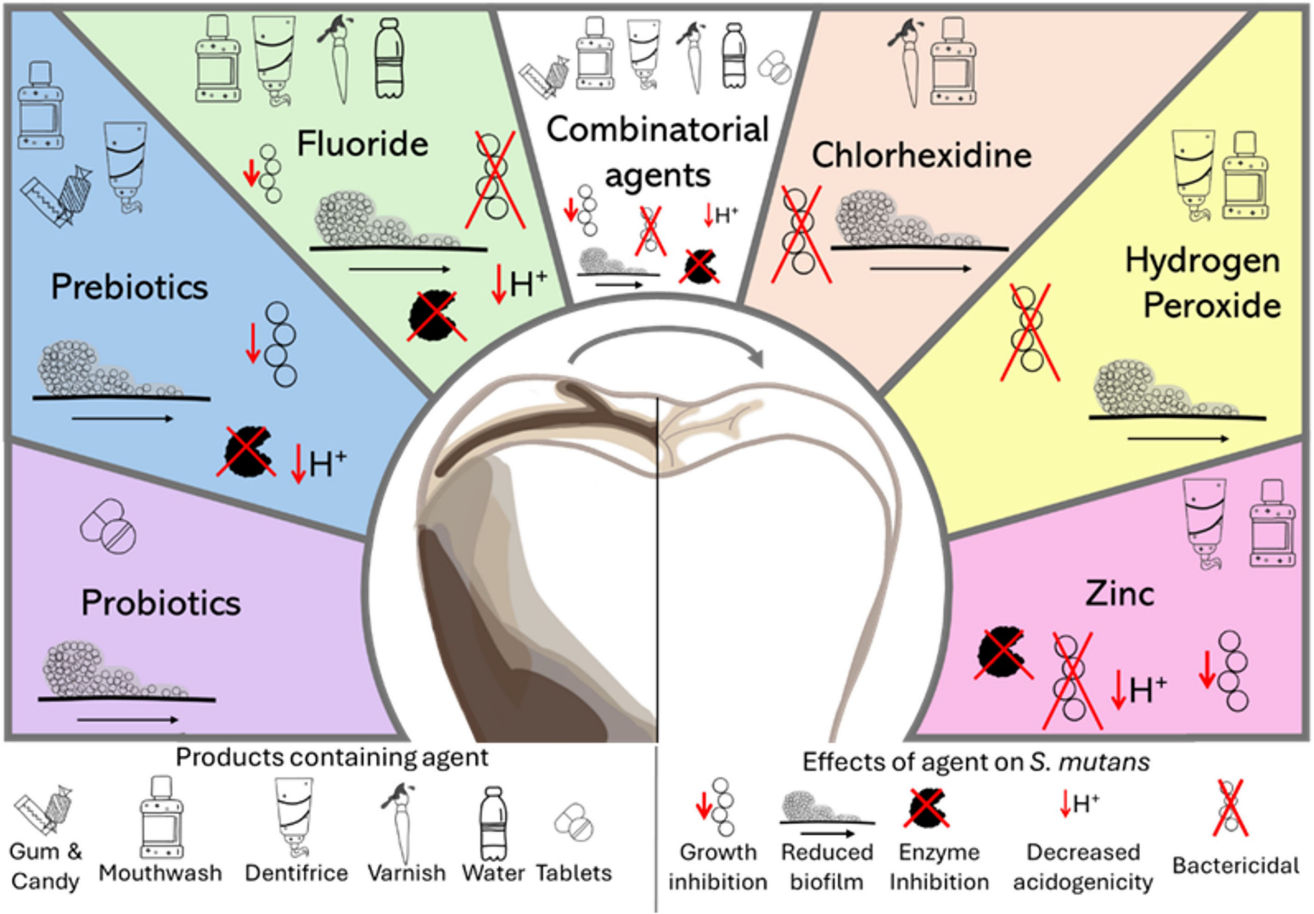
Oral hygiene agents, their means of administration and effect against *Streptococcus mutans*. Oral hygiene agents are depicted, indicating products that contain the various agents and the effect of these agents against the caries associated bacterium *Streptococcus mutans*. Most agents are available in more than one type of product, and many of them have been shown to inhibit *S. mutans* by interfering with multiple aspects of virulence.

In closing this mini-review, it should be acknowledged that in discussing each of the agents, our focus was on their abilities to specifically target *S. mutans*. However, dental caries is a multifactorial disease that occurs in an oral environment harboring hundreds of species of microbes, the metabolisms and virulence factors of each contributing to the status of health or disease. The future design and development of anti-caries agents must consider that caries occurs as a polymicrobial disease; expanding studies to understand how these agents impact other oral microbes will enhance our ability to inhibit disease (Zhang et al., 2022). As the combinatorial therapy is largely an emerging strategy, future studies are likely to elucidate the molecular mechanisms that yield successful pairings and to consider host factors such as salivary flow, food consumption, and hygiene that are likely to impact the efficacy of the agents. Each of the agents discussed in this review offers distinct mechanisms to inhibit the ability of *S. mutans* to foster a cariogenic microbiome, ranging from metabolic inhibition to ecological modulation. However, it is possible that using these agents together in combination therapy may yield enhanced benefits and better reflect real-world oral hygiene practices. Continued research into these interactions is essential for developing novel oral care products and for better utilizing existing agents to promote long-term oral health. **Figure 1** summarizes the administration and anti-bacterial effects of each of the agents described.
